# HiCMC: High-Efficiency Contact Matrix Compressor

**DOI:** 10.1186/s12859-024-05907-2

**Published:** 2024-09-10

**Authors:** Yeremia Gunawan Adhisantoso, Tim Körner, Fabian Müntefering, Jörn Ostermann, Jan Voges

**Affiliations:** 1 Institut für Informationsverarbeitung and L3S Research Center, Leibniz University Hannover, Hannover, Germany; 2 CIMA University of Navarra, Pamplona, Spain; 3 IdiSNA, Pamplona, Spain

**Keywords:** Contact matrix, Hi-C, 3C, Compression

## Abstract

**Background:**

Chromosome organization plays an important role in biological processes such as replication, regulation, and transcription. One way to study the relationship between chromosome structure and its biological functions is through Hi-C studies, a genome-wide method for capturing chromosome conformation. Such studies generate vast amounts of data. The problem is exacerbated by the fact that chromosome organization is dynamic, requiring snapshots at different points in time, further increasing the amount of data to be stored. We present a novel approach called the High-Efficiency Contact Matrix Compressor (HiCMC) for efficient compression of Hi-C data.

**Results:**

By modeling the underlying structures found in the contact matrix, such as compartments and domains, HiCMC outperforms the state-of-the-art method CMC by approximately 8% and the other state-of-the-art methods cooler, LZMA, and bzip2 by over 50% across multiple cell lines and contact matrix resolutions. In addition, HiCMC integrates domain-specific information into the compressed bitstreams that it generates, and this information can be used to speed up downstream analyses.

**Conclusion:**

HiCMC is a novel compression approach that utilizes intrinsic properties of contact matrix, such as compartments and domains. It allows for a better compression in comparison to the state-of-the-art methods. HiCMC is available at https://github.com/sXperfect/hicmc.

## Introduction

The human genome provides critical insights into a wide range of biological processes. In recent decades, advances in high-throughput sequencing technologies have reduced the costs associated with genome sequencing [1]. This cost reduction has enabled large-scale studies such as genome-wide association studies [2] and the development of the concept of polygenic risk scores [3]. These studies involve the systematic analysis of hundreds of thousands of genetic variants associated with specific traits or diseases. They unravel many complex interactions between genotypes and phenotypes.

**Fig. 1:**
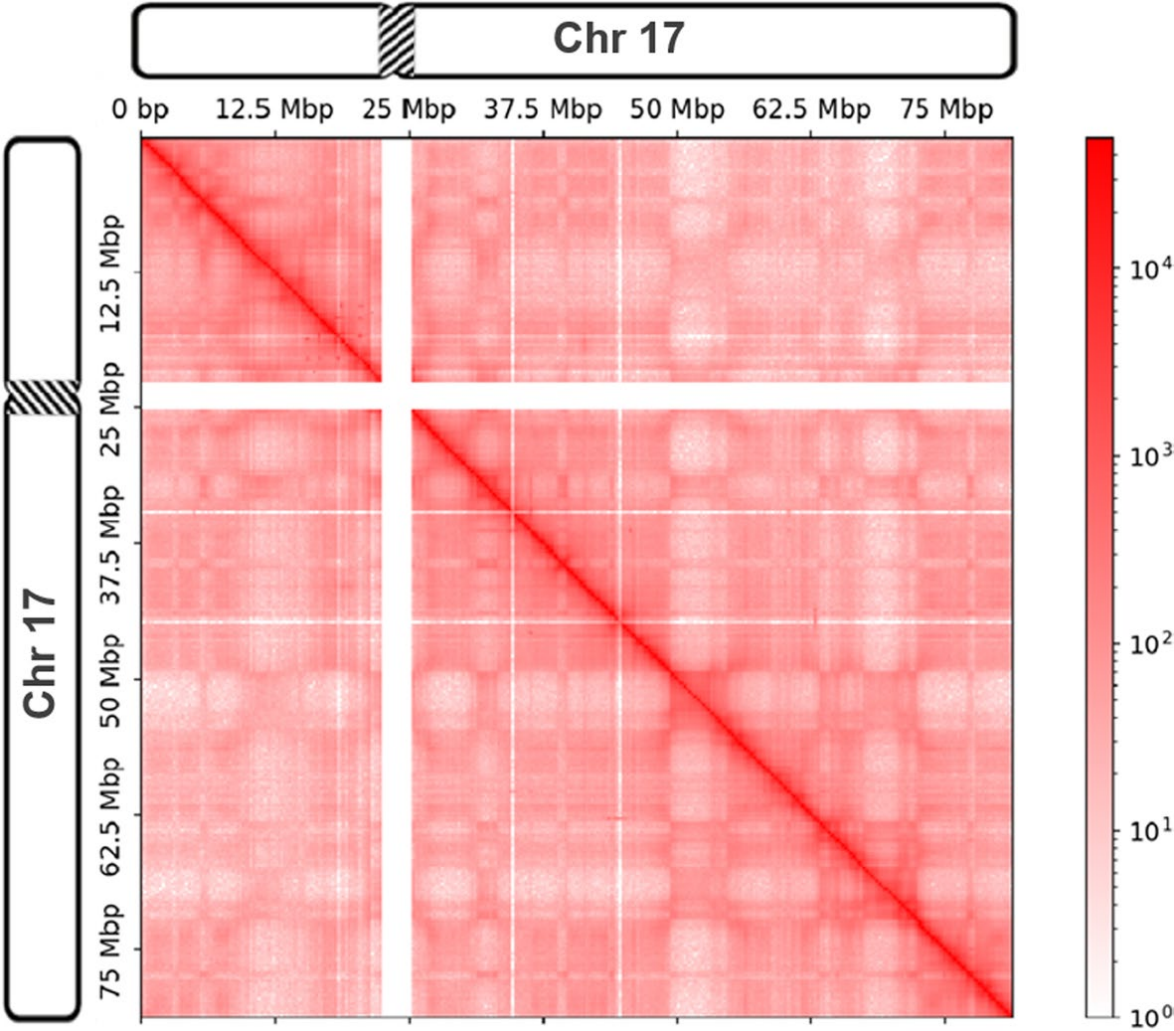
An example of an intra-chromosomal (*cis*) contact matrix of human chromosome 17. Interactions are highly correlated with spatial proximity, and hence, highly-interacting regions are colored in dark red while regions with a low amount of interactions are colored in brighter shades of red. Note that the contact matrix is sparse, symmetrical and contains regions with no interactions, shown as white rows and columns

Simultaneously, the advances in high-throughput sequencing technologies have spurred advances in the field of epigenetics [4], i.e., the study of biological processes that do not involve alterations directly in the underlying DNA sequence, but with regard to other genetic features such as spatial chromosome organization and DNA methylation. One of the most important findings has been the critical role of spatial chromosome organization in biological functions such as replication, regulation, and transcription [5, 6]. One way to analyze the three-dimensional structure of chromosomes is through chromosome conformation capture (3C) [7], a ligation-based approach that captures the interactions between pairs of loci. 3C successors such as Hi-C and Micro-C [8–11] are able to capture genome-wide interactions between all possible pairs of loci of all chromosomes simultaneously and with much higher resolution. Hi-C and Micro-C allow the identification of long-range interactions and provide insights into finer chromosomal structures such as topologically associating domains (TADs) and loop domains [12, 13]. Figure 1 shows an example of a so-called intra-chromosomal (*cis*) contact matrix as a result of a Hi-C experiment. In the figure, highly interacting regions are colored in dark red, while regions with fewer interactions are colored in lighter shades of red. From the figure, it can hence be seen, e.g., by the dark red diagonal, that interactions are highly correlated with spatial proximity. Each row and column of the contact matrix represents a region of a specific size. The size of the regions is referred to as resolution. With high-resolution contact matrices it is hence possible to reveal finer structures.

Hi-C experiments generate enormous amounts of data, especially if they are performed at high resolution, i.e. counting interactions at a small granularity. In addition, recall that the three-dimensional organization of chromosomes is dynamic. It changes over time and exhibits cell type specificity. This problem is exacerbated as the field of genomic research, particularly chromosome conformation capture, is rapidly moving toward larger and more complex experiments, including single-cell Hi-C studies [14, 15] that contain tens of thousands of cells. Thus, comprehensive analyses require the examination of chromosome organization across multiple temporal snapshots, compounding the challenge of data volume.

In the field of sequencing data, attempts to represent and compress the data began with the ACII-based FASTQ [16] format for unaligned data, which can be compressed with the general–purpose compressor gzip [17]. Similarly, aligned data can be stored in the ASCII-based SAM [18] format, but is commonly stored in the binary BAM [18] format. Recently, the community efforts to further improve the compression of sequencing data led to the development of CRAM 3.1 [19]. In parallel, the Moving Picture Experts Group (MPEG), an ISO and IEC working group, published its first international standard (ISO/IEC 23092, known as MPEG-G) for sequencing data [20]. While many attempts have been made for sequencing data, a common format for representing contact matrix data with a dedicated compressor capable of handling large data sets is lacking [21]. Several formats have been developed to provide efficient storage of Hi-C data, such as hic [22] and butlr [23]. Later, cooler [24], based on the HDF5 [25] format, was introduced. The HDF5 format provides flexible organization of multidimensional arrays, support for random access, and data compression based on Zlib [26] and sZIP [27]. Cooler takes advantage of the sparsity and symmetry properties of contact matrices by storing and stores these in Coordinate List (COO) representation. However, the performance of HDF5 compression is inferior compared to modern general purpose compression methods such as the Lempel–Ziv–Markov chain algorithm (LZMA) [28], Zstandard (ZSTD) [29], and bzip2 [30]. Also it does not exploit prior information about chromosomal structures found in the contact matrix. In contrast to the aforementioned formats, Contact Matrix Compressor (CMC) [31] improves compression performance by exploiting several properties of the contact matrix, including the correlations between genomic distance and interactions, unalignable regions, and symmetry. While CMC improves compression, it does not take advantage of the finer structures found in intra-chromosomal contact matrices, such as compartments and TADs. In this work, we present a novel approach, HiCMC, for contact matrix compression. Better performance is achieved by modeling structures in the intra-chromosomal contact matrix.

## Methods

**Fig. 2:**
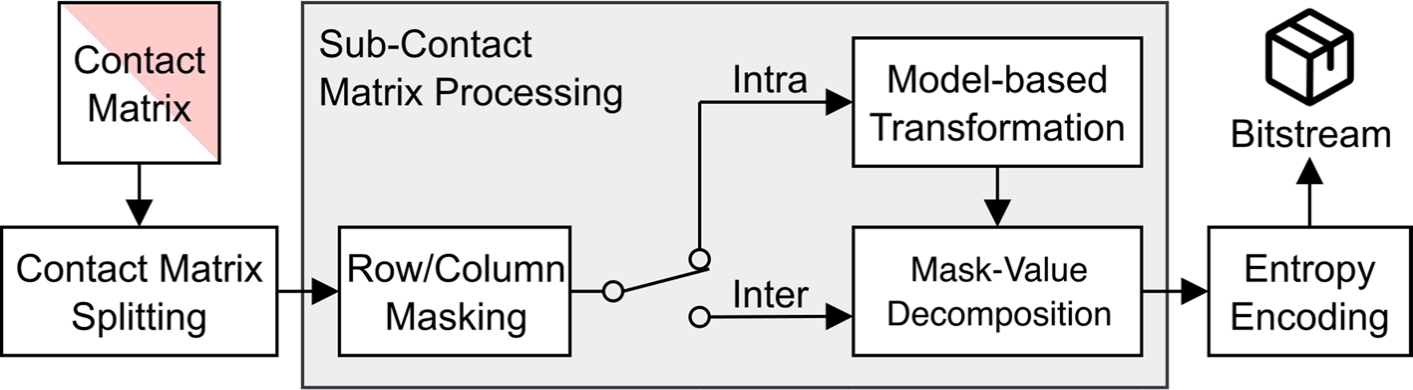
The HiCMC compression pipeline consists of splitting the genome-wide contact matrix into intra- and inter-chromosomal contact matrices, row/column masking, model-based transformation, row binarization, and entropy coding. The type of input sub-contact matrix determines whether Intra or Inter is used

Our approach HiCMC is a major extension of CMC [31]. It comprises splitting the genome-wide contact matrix into intra- and inter-chromosomal sub-contact matrices, row and column masking, model-based transformation, row binarization, and entropy coding as shown in Fig. 2. The key idea of CMC is to transform contact matrix values so that in each row of the matrix the number of bits required for each value, i.e. the magnitude of the values, is similar. This facilitates more efficient entropy coding. The main drawback of CMC is that it does not account for structures that exist in an intra-chromosomal contact matrices, such as compartments and domains, which are highly interacting with themselves. These structures cause the interactions in certain regions of the contact matrix to be lower or higher than the expected interactions based on the distance. HiCMC improves intra-chromosomal contact matrix compression by modeling the aforementioned structures in a step called model-based transformation. For the inter-chromosomal contact matrix, no changes were made to the compression pipeline. These processes will be discussed in the following sections.

**Fig. 3:**
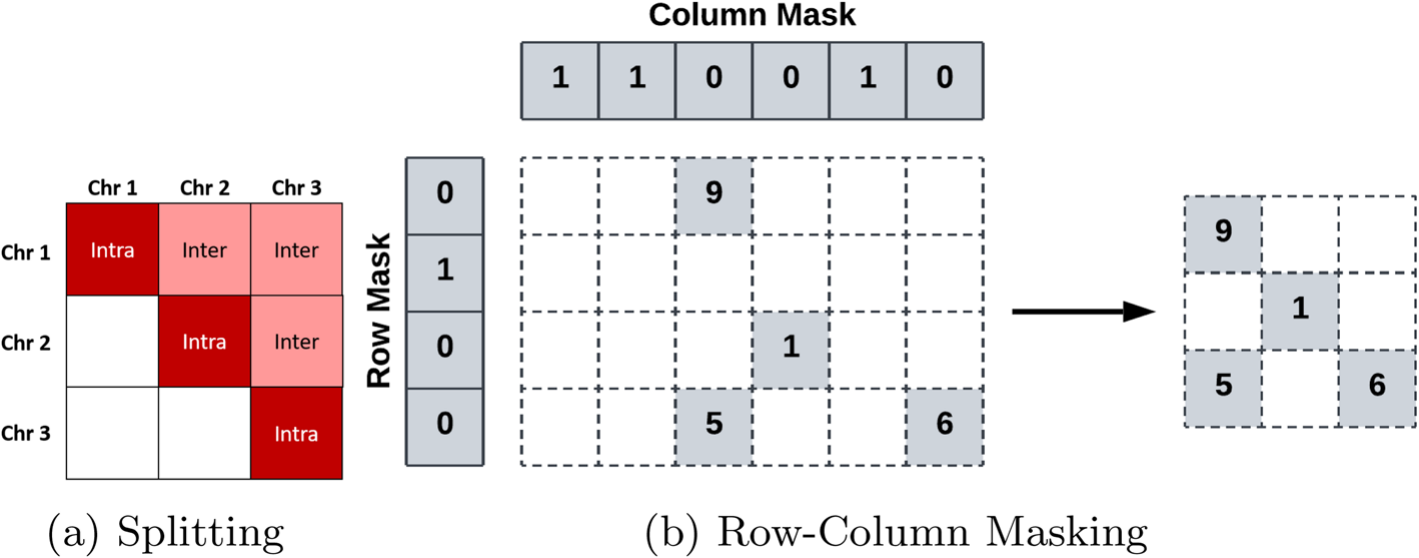
Splitting and masking processes of HiCMC. (a) The contact matrix is divided into two different sub-contact matrices based on chromosome-chromosome interactions: intra-chromosomal (Intra) and inter-chromosomal (Inter) sub-contact matrix. We only store the sub-contact matrices that are in the main diagonal and upper triangle of the matrix. (b) The masking process works by marking empty rows/columns in the corresponding mask (left) and then removing them from the original matrix to construct the masked matrix (right)

### Split contact matrix

The first step in the compression pipeline is to divide a chromosome-wide contact matrix into chromosome-chromosome interaction matrices, hereafter referred to as sub-contact matrices. Due to the symmetry of contact matrices, only sub-contact matrices lying within the upper triangle need to be stored. The contact matrix after splitting is shown in the Fig. 3a.

### Row-column masking

To remove redundant information in sub-contact matrices efficiently, we next remove unalignable regions [31] — rows and/or columns with no interactions —  by first marking the rows and columns with the binary masks (see Fig. 3b). The mask entry is set to 1 for the corresponding rows or/and columns containing only zeros, otherwise it is set to 0. The pipeline is branched differently depending on the type of sub-contact matrix: intra- or inter-chromosomal sub-contact matrix.

### Model-based transformation

The diagonal transformation of CMC assumes that the values in a diagonal of the contact matrix are of approximately similar magnitude. This transformation reflects the observation that the chromosomal interactions serve as an approximation of spatial distance [8]. By placing the entries from the same diagonal in a row in the new matrix, the number of bits required to represent the values in each row can be reduced. However, due to structures such as A/B compartments or TADs, it provides only a basic approximation of the interactions. Interactions within compartments and TADs are enriched, but an abrupt drop in interactions is observed for inter-compartments and inter-TADs [9].

**Fig. 4:**
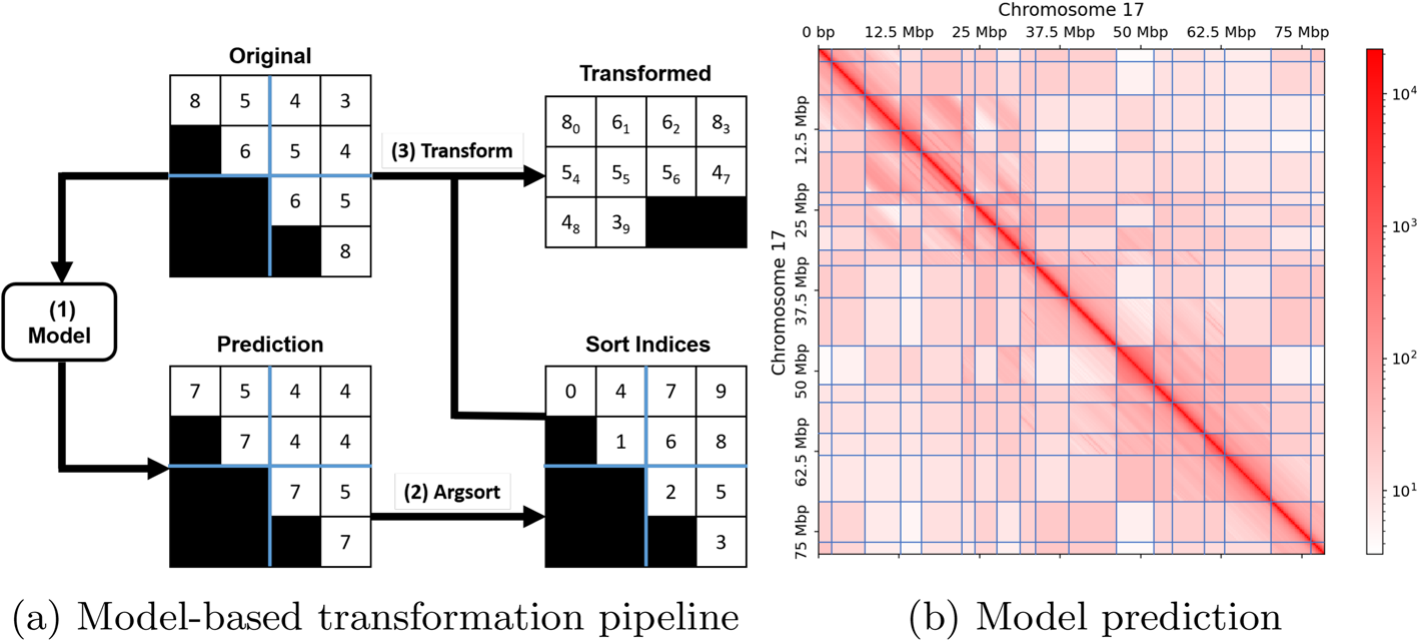
Overview of the model-based transformation pipeline and model prediction. (**a**) The model-based transformation pipeline creates models based on the entries of the sub-contact matrix and domain boundaries, and uses these models to generate predictions (step 1). The pipeline then sorts these predictions in magnitude order, resulting in sorting indices (step 2). Finally, the pipeline rearranges the original interactions according to these indices, starting from the top left to the bottom right (step 3). (**b**) An example of the model prediction derived from Fig. 1. Domain matrices modeled using the genomic distance function, characterized by color gradients such as the domain on the main diagonal, can be distinguished from those modeled using a constant domain value. The blue lines represent the domain boundaries determined by a TAD caller

To overcome the limitations of existing approaches, we propose a novel model that represents a sub-contact matrix as a set of rectangular intra- and inter-domain matrices. Specifically, we model intra-domain matrices using genomic domain information and inter-domain matrices using a constant value. To derive the domain matrices, we must first determine the domain boundaries of domains using a TAD caller. Figure 4b illustrates an example of the domain boundaries predicted by a TAD caller, denoted by the blue lines. Constructing and efficiently encoding this model is crucial, and various methods can be explored. Moreover, biases in visibility across regions of a chromosome, such as GC-rich regions and regions that are difficult to map, can affect boundary prediction. To improve model accuracy, we construct the model from a balanced matrix, thereby removing experimental bias introduced in the experiments.

We divide the sub-contact matrix into two types of rectangular regions, which we refer to as domain matrices: inter-domain matrices and intra-domain matrices. For intra-domain matrices, we model the entries based on the function of genomic distance $f(j - i)$, where *i* and *j* represent the row and column indices, respectively. In contrast, inter-domain matrices are modeled using a single constant value, which is advantageous because average interactions for certain domains are roughly constant and no longer correlate with genomic distance. The decision on how to model each domain matrix depends on the statistical properties of the domain matrix entries and the corresponding threshold, both of which are encoding parameters. Specifically, we compute the standard deviation of the non-zero entries to determine the domain matrix type. We encode this decision as a binary matrix called “domain classes”, where each entry represents the type of domain matrix for each domain.

We transform the original sub-contact matrix by sorting its entries based on the modeled matrix entries, as shown in Fig. 4a. This process involves three steps: First, we model the domain matrices and predict the entries of the contact matrix based on our domain matrices. Next, we determine the sorting indices from these predictions. Finally, we sort the contact matrix by placing each entry of the original matrix into its corresponding index. Figure 4b illustrates the predicted domain boundaries and the modeled matrix entries.

In detail, the genomic distance function for intra-domain matrices is implemented as a “distance table”, where each entry represents the average value of intra-domain matrix entries at a given genomic distance. The table is organized with columns representing specific genomic distances and rows representing specific domains, grouping values of similar magnitude together. The entries in both the sub-contact matrix and the domain matrix that lie on the same diagonal share the same genomic distance. For each domain matrix, we compute the average value of a particular diagonal and append it to the distance table. This organization enables efficient entropy coding, resulting in a higher compression ratio.

$$\begin{aligned} \begin{array}{c} \left[ \begin{array}{c|c} \begin{array}{cc} c_{11} & c_{12} \\ 0 & c_{22} \end{array} & \begin{array}{cc} c_{13} & c_{14} \\ c_{23} & c_{24} \end{array} \\ \hline \begin{array}{cc} 0 & 0 \\ 0 & 0 \end{array} & \begin{array}{cc} c_{33} & c_{34} \\ 0 & c_{44} \end{array} \end{array} \right] \\ \text {Contact Matrix}\:C \end{array} \begin{array}{c} \left[ \begin{array}{c|c} \begin{array}{cc} {{D_{00}}}\\ & \end{array} & \begin{array}{cc} {{D_{01}}}\\ & \end{array} \\ \hline \begin{array}{cc} {{D_{10}}}\\ & \end{array} & \begin{array}{cc} {{D_{11}}}\\ & \end{array} \end{array} \right] \\ \text {Domains Matrices}\:D \\ \end{array} \quad \rightarrow \quad \begin{array}{c} \begin{array}{cccc} 0 & 1 & 2 & 3 \\ \hline d^{(0)}_{00} & d^{(1)}_{00} & d^{(2)}_{01} & d^{(3)}_{01} \\ d^{(0)}_{11} & d^{(1)}_{01} & 0 & 0 \\ 0 & d^{(1)}_{11} & 0 & 0 \end{array}\\ \text {Distance Table}\:T\end{array} \end{aligned}$$

To illustrate this process, let us consider a $4 \times 4$ contact matrix *C* with entries $c_{ij}$ at position (*i*, *j*). We assume that the domain matrices have a size of $2 \times 2$ and are indexed with (*ab*). Due to the symmetrical property of the contact matrix, its lower triangular entries are zero. Each column of the distance table stores the average entries of all domains for a specific genomic distance *k*. We compute the entries of the distance table $d^{(k)}_{ab}$ by averaging all contact matrix entries $c_{ij}$ that belong to a domain matrix $D_{ab}$ at a distance of *k*:

$$\begin{aligned} d^{(k)}_{ab} = \mathbb {E} \bigl [c_{ij}\bigr ], \forall {c_{ij}\in D_{ab}} \wedge k = j-i \wedge c_{ij} \ne 0 \end{aligned}$$

where $\mathbb {E} \bigl [\cdot \bigr ]$ denotes the averaging operation.

For inter-domain matrices, We store the average interactions of each domain in a matrix called “domain values”, as these matrices are modeled using a single constant value. The “domain value” matrix has the same shape as the “domain classes” matrix.

Based on the domain classes, the distance table, and the domain values, we predict the entries of the sub-contact matrix. For a domain modeled as a function of genomic distance, we retrieve the entries at a given genomic distance from the distance table. Otherwise, we set all entries of the corresponding domain matrix to the domain value of the corresponding domain retrieved from the domain values matrix, resulting in a predicted domain matrix with a uniform value.

Figure 4b illustrates an example of a predicted sub-contact matrix. The model used for prediction must be included in the compressed payload, which introduces an overhead. This leads to a trade-off between the quality of our model and the compression performance. To mitigate the overhead, we reduce the floating-point precision of both the distance table and the domain values, thereby striking a balance between model quality and compression efficiency. It is important to note that the floating-point precision reduction does not render our compression method lossy, as the prediction is used to sort the original sub-contact matrix, and the reduction occurs prior to the prediction step in the encoding process, thereby preserving all information.

For the model-based transformation, our primary goal is to infer the sorting order based on the prediction as similar as possible compared to the sorting order based on the original contact matrix, i.e. to predict the underlying relative differences between contact matrix entries (as measured by Spearman’s rank correlation) rather than to predict the magnitudes (which would be similar to minimizing the mean square difference). Furthermore, minimizing the absolute differences would introduce significant overhead for long-range interactions (i.e., entries for which the difference between row and column IDs is large) due to random ligation. We evaluate the quality of the model by examining the overall reduction in size, rather than directly assessing the model’s sorting using Spearman’s rank correlation between the original and predicted matrices. This approach is necessary because of the complex relationship between the model-based transformation and the entropy coding step.

### Mask-value decomposition

Following the application of the model-based transformation, we decompose the transformed sub-contact matrix using mask value decomposition. Unlike row binarization in CMC, this decomposition yields comparable compression performance with a significantly simpler process. Mask-value decomposition separates the sub-contact matrix into two components: a binary matrix indicating the positions of non-zero entries, and a separate array containing the corresponding non-zero values. We refer to these two components as the sub-contact matrix mask and the sub-contact matrix values, respectively.

### Entropy coding

In total, four payloads are required for the model: the domain boundaries, the domain classes, the domain values, and the distance table. The domain boundaries can be represented as a one-dimensional binary array indicating the presence or absence of a boundary for each bin. It can be efficiently encoded using binary run-length encoding [31], since long sequences of zeros (indicating the absence of a boundary) are expected.

Both the domain classes and the sub-contact matrix mask are binary matrices. Since there are many 1’s along the main diagonal of the matrix, it is first transformed using the diagonal transformation [31] and then compressed using an encoder conforming to the Joint Bi-level Image Experts Group (JBIG) standard (ISO/IEC 11544 [32]), specialized for lossless compression of bi-level (i.e., binary) images. It takes advantage of the spatial correlation of neighboring binary pixels.

The domain values matrix is also transformed using the diagonal transformation, as higher values tend to be placed along the main diagonal. Both the domain values and the distance table matrix are encoded by serializing them into an array, which is also compressed using fpzip [33] with a certain floating point precision, which controls the quality of the model as mentioned in Sect. 2.3. Finally, the sub-contact matrix values are compressed using the prediction by partial patching (PPM) [34]-based technique PPMd [35].

## Results and discussion

For the evaluation, we use the dataset published by Rao et al. [9] and available under the NCBI accession code GSE63525. The dataset consists of contact matrices from human cell lines (GM12878, HMEC, HUVEC, IMR90, K562, KBM7, and NHEK) and mouse B-lymphoblasts (CH12) at multiple resolutions. To compare the size of compressed data with cooler, we convert the data to cooler format using the hic2cool tool. The dataset is described in Table 1. Our method, HiCMC, is available at https://github.com/sXperfect/hicmc.

**Table 1:** The dataset used for evaluation consists of contact matrices at multiple resolutions from different cell lines and based on different approaches. We focus on the intra-chromosomal sub-contact matrices

Cell line	hic [GB]	Cooler [GB]	cooler (intra) [GB]
CH12	8.58	1.90	0.43
GM12878 (Insitu-DpnII)	6.83	1.37	0.44
GM12878 (Insitu-Primary)	31.86	9.64	1.77
GM12878 (Insitu-Replicate)	29.06	8.78	1.63
HMEC	7.08	1.46	0.20
HUVEC	8.87	1.92	0.27
IMR90	12.77	2.72	0.44
K562	12.08	2.61	0.37
KBM7	13.91	3.19	0.33
NHEK	11.34	2.55	0.62

**Table 2:** The resolution-specific parameter sets used by our compression pipeline: Window Size, Threshold, Distance Table Precision, Domain Values Precision, and Balancing Weights Precision. The parameter values are optimized using the Tree-structured Parzen Estimator (TPE) algorithm

Resolution	5 kb	10 kb	25 kb	50 kb	100 kb	250 kb
Window Size	32	16	8	4	4	4
Threshold	5.0	7.5	13.5	15.0	45.0	45.0
Distance Table Precision	10	10	10	10	10	10
Domain Values Precision	10	10	10	11	11	18
Balancing Weights Precision	12	10	10	12	12	12

**Fig. 5:**
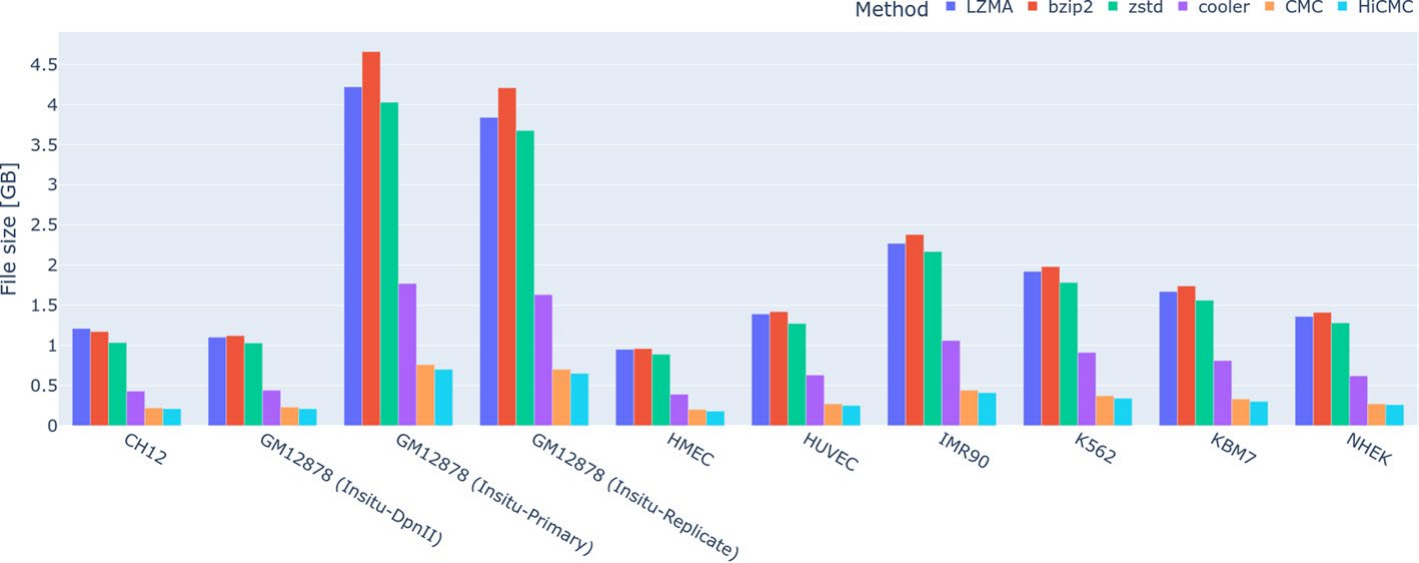
Absolute payload sizes of the compressed intra-chromosomal contact matrices. HiCMC outperforms CMC, cooler, LZMA, ZSTD, and bzip2 across all resolutions and cell lines

**Table 3:** Absolute payload sizes of compressed intra-chromosomal contact matrices across cell lines using different methods, in gigabytes, as visualized in Fig. 5. Methods are sorted from left to right by year of publication and Gradual Improvement shows the improvement over the previously published method

Method	LZMA	bzip2	ZSTD	Cooler	CMC	HiCMC
CH12	1.21	1.17	1.03	0.43	0.22	0.21
GM12878 (Insitu-DpnII)	1.10	1.12	1.03	0.44	0.23	0.21
GM12878 (Insitu-Primary)	4.22	4.66	4.03	1.77	0.76	0.70
GM12878 (Insitu-Replicate)	3.84	4.21	3.68	1.63	0.70	0.65
HMEC	0.95	0.96	0.89	0.39	0.20	0.18
HUVEC	1.39	1.42	1.27	0.63	0.27	0.25
IMR90	2.27	2.38	2.17	1.06	0.44	0.41
K562	1.92	1.98	1.78	0.91	0.37	0.34
KBM7	1.67	1.74	1.56	0.81	0.33	0.30
NHEK	1.36	1.41	1.28	0.62	0.27	0.26
Average size	1.99	2.11	1.87	0.87	0.38	0.35
Improvement w.r.t. cooler	$-$129.34%	$-$142.23%	$-$115.42%	0.00%	56.39%	59.61%
Gradual improvement	N/A	$-$5.62%	11.07%	53.58%	56.39%	7.39%

**Fig. 6:**
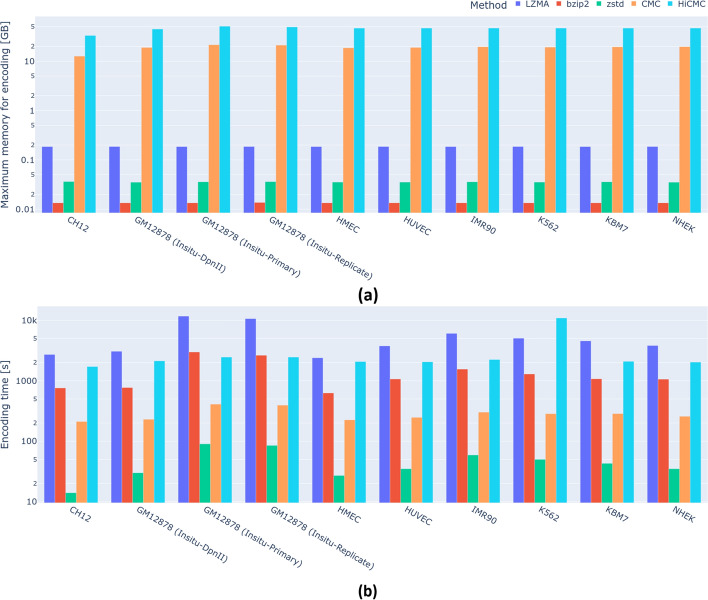
Encoding complexity of all methods. (**a**) Maximum memory used by each method during the encoding process, corresponding to the memory used to compress contact matrices at 5 kb resolution. (**b**) Total encoding time to compress all chromosomes and all resolutions of a given cell line

**Fig. 7:**
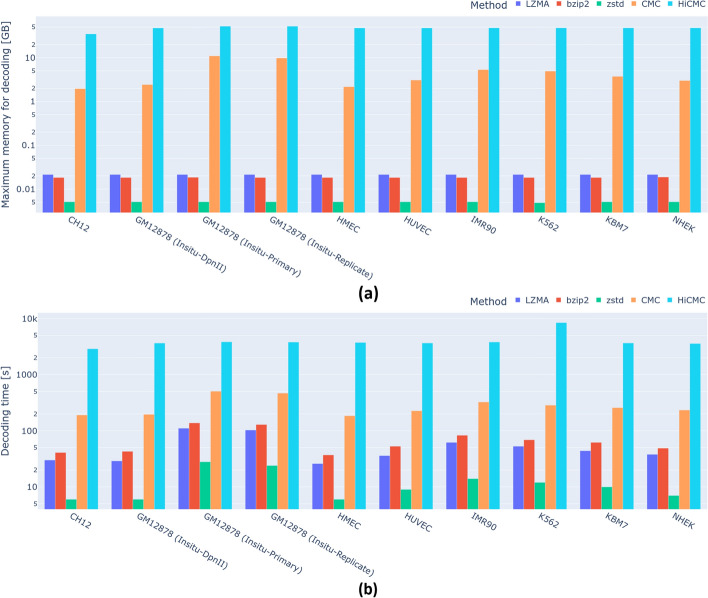
Decoding complexity of all methods. (**a**) Maximum memory used by each method during the decoding process, corresponding to the memory used to compress contact matrices at 5 kb resolution. (**b**) Total decoding time to compress all chromosomes and all resolutions of a given cell line

Since our approach extends the compression pipeline of CMC for intra-chromosomal sub-contact matrices, we limit the comparison to the intra-chromosomal contact matrices. As a pre-processing step before the actual compression, we predict the domain boundaries for each intra-chromosomal contact matrix using TAD callers based on the insulation score [36] that is an integral component of cooltools [37]. The contact matrices are balanced using the Knight-Ruiz normalization (KR) [38] algorithm. The compression process in HiCMC is controlled by five encoding parameters: window size, threshold, distance table precision, domain value precision, and balancing weight precision. The domain border is determined by the insulation score, which aggregates interactions in a sliding window along the diagonal. The insulation score has a window size parameter that specifies the size of the previously mentioned sliding window. The domain table precision, domain values precision, and balancing weights precision specify the precision of the floating point for encoding the corresponding payloads using fpzip. Last, the threshold determines the threshold value used to select a mode for the domain: representing a domain with its average or as a function of genomic distance. For all resolutions and cell lines, the statistical characteristic of the domain matrix is computed based on the standard deviation of all entries of the corresponding domain matrix. The parameters are optimized using the TPE algorithm [39, 40] and the parameters are valid across different resolutions and cell lines. The resolution-specific parameter sets are described in Table 2. CMC does not create a model and therefore no hyperparameter optimization is performed on the CMC. For transcoding purposes, the cooler format is the easiest to work with. For compression with LZMA, ZSTD, and bzip, the contact matrices are converted to the GInteractions [41] format using the HiCExplorer [42] tool. Subsequently, the matrices are compressed using their corresponding software and default parameters. Both CMC and HiCMC can take cooler as input directly. Since we mainly use cooler as input for all other methods, we exclude the run time and memory usage for cooler.

As shown in Fig. 5 and Table 3, HiCMC outperforms all other methods in terms of compression for intra-chromosomal contact matrices across all resolutions and cell lines. Interestingly, ZSTD is faster and uses less memory than the other general-purpose compression methods while compressing the data better. HiCMC exhibits a gradual (i.e. w.r.t to CMC) improvement of 7.39%. Compared to the de facto standard cooler, HiCMC shows a compression improvement of 59.61%. Assuming the use case of contact matrix storage in single cell experiments, the estimated space saving of HiCMC w.r.t. to cooler is approximately 0.52 GB per cell, providing a significant advantage since such experiments typically contains tens of thousands of cells. We show that our method works well on both normal and abnormal cells from a patient with myelogenous leukemia (K562). While both HiCMC and CMC show superior performance, the encoding complexity of both methods is higher compared to the other methods due to the transformation performed on the dense matrix form, which is quite large, especially for the contact matrix at 5 kb resolution. This complexity analysis is shown in Fig. 6 for encoding process and Fig. 7 for decoding process.

**Fig. 8:**
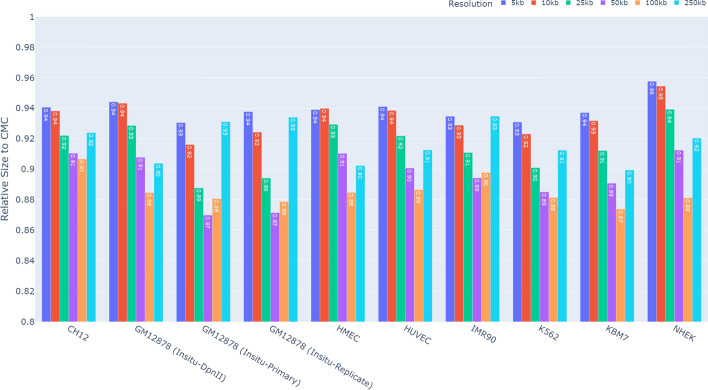
Relative size of the compressed HiCMC payload in comparison to that of CMC. HiCMC outperforms CMC across all resolutions and cell lines

Since both HiCMC and CMC dominate all other methods in terms of compression performance, and for simplicity, we compare HiCMC to CMC for each resolution over all cell lines shown in Fig. 8. Because the size of the domains is relatively large, it is most efficient to compress data at medium resolution (25 kb to 100 kb). The compression of the contact matrix can be further improved by experimenting with other TAD callers. Note that domain information, such as the information produced by the TAD caller, is embedded in bitstreams produced by HiCMC. This way, further downstream analysis that relies on the estimation of domain boundaries or TAD caller can be accelerated by exploiting this domain information, for example if only information about a specific TAD is of interest.

**Fig. 9:**
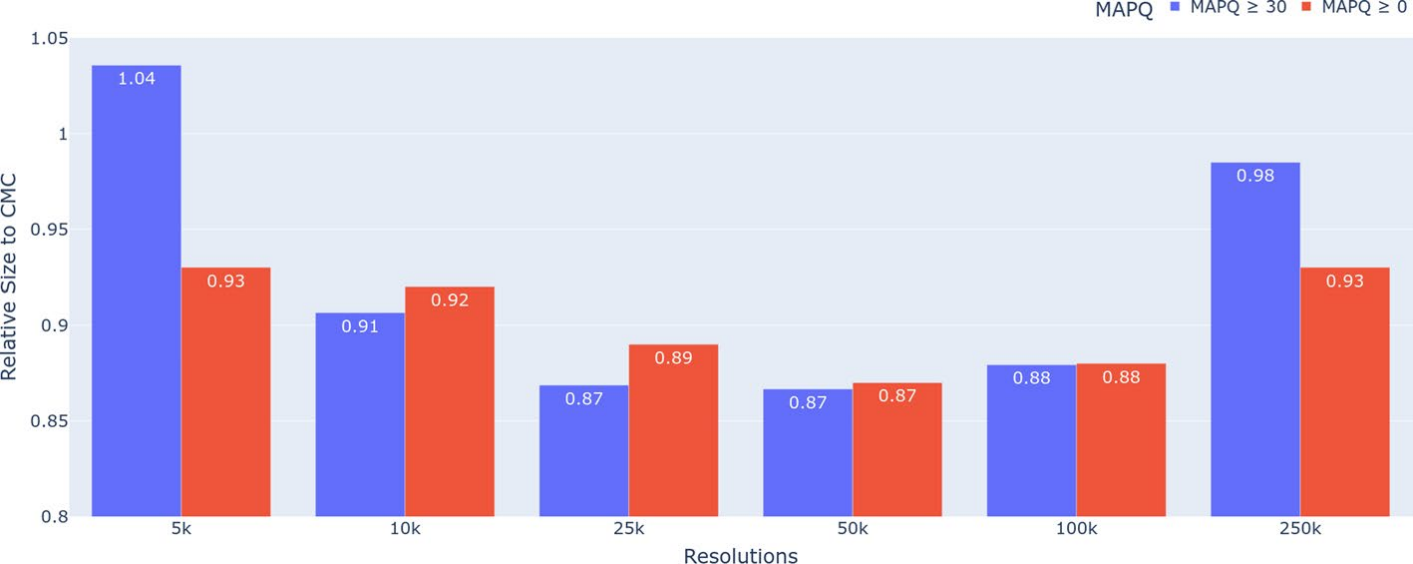
Relative size of the compressed HiCMC payload in comparison to that of CMC on GM12878 (Insitu-Primary) to assess the compression performance at different MAPQs. The contact matrices are compressed using parameters optimized for MAPQ $\ge$ 0

To evaluate the performance of HiCMC at different MAPQ values, we performed an additional experiment by compressing GM12878 (Insitu-Primary) contact matrices with MAPQ $\ge$ 0 and MAPQ $\ge$ 30 as shown in Fig. 9. We chose the parameters optimized for MAPQ $\ge$ 0 as shown in Table 2. For resolutions between 10 kb and 100 kb, the compression ratio is comparable. We believe that the threshold should be optimized for MAPQ $\ge$ 30 to match the less noisy data.

**Fig. 10:**
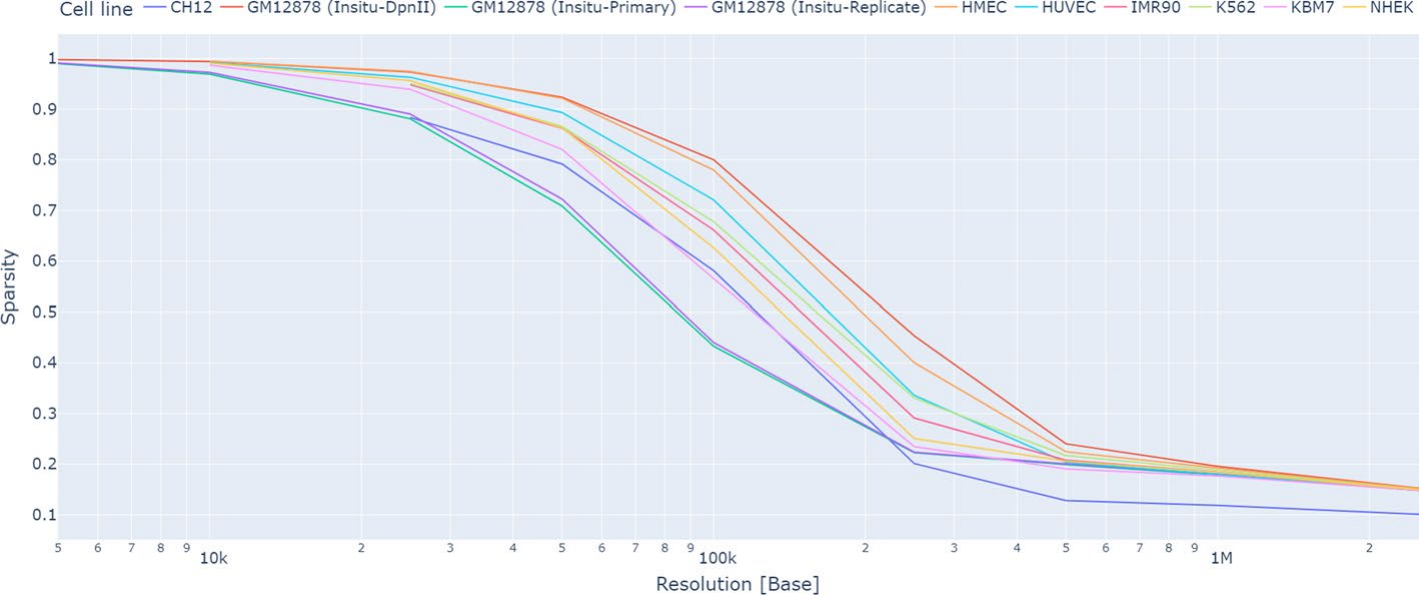
The contact matrix sparsity is directly related to its resolution. When the resolution exceeds 20 kb (i.e., a lower value), the sparsity exceeds 90%. Therefore, we propose that developing more efficient sparse matrix transformation and encoding methods would be beneficial for encoding high-resolution contact matrices

Although HiCMC offers the best compression performance, it is computationally expensive. Some factors that contribute to the increased coding time and memory usage compared to CMC are the predicted contact matrix and the sorting step, both of which are part of the encoding and decoding process. This increase is further exacerbated by dense matrix operations, which approximately double the computational cost. As shown in Fig. 10 , there is a direct relationship between sparsity and resolution for contact matrix data. For high resolution data (10 kb or lower value), the sparsity is 90% or higher, leaving room for a potential 10x improvement in terms of memory and speed. In addition, the model-based transformation, relies heavily on the sorting process, which is highly parallelizable. By using a parallelizable sorting method, especially on a GPU, we can substantially reduce the runtime. Our experiments solely aims to demonstrate the compression performance of our approach, leaving the computational optimization to future work. Furthermore, in the compressed payload high-resolution contact matrix, a significant proportion of the storage is allocated to store the coordinates of observed interactions, rather than the actual interaction data itself. To further improve compression performance for high-resolution contact matrices, we believe that the development of methods that exploit sparsity would be beneficial in improving both compression performance and coding complexity.

## Conclusions

We have presented HiCMC, a specialized model-based compressor for encoding contact matrices. It outperforms the state of the arts, including cooler, general-purpose compressors such as LZMA, ZSTD, and bzip2, as well as the specialized contact matrix compressor CMC. HiCMC outperforms CMC by approximately 8% and is superior to other approaches for encoding intra-chromosomal contact matrices by at least 50%. HiCMC achieves better performance by exploiting the underlying properties of contact matrices, such as their symmetry and correlations between genomic distance and interactions, as well as further hierarchical structures of chromosomal organization reflected in the matrices, in particular TADs. HiCMC exploits these properties by constructing appropriate models and using them to predict the values of the associated contact matrices. HiCMC determines the domain boundaries based on the insulation score, but other TAD callers can be experimented with to improve compression performance.

## Data Availability

The HiC data of all cell lines used in this study are available under the NCBI accession code GSE63525. The HiCMC source code is available at https://github.com/sXperfect/hicmc. The hic2cool tool used for the conversion from hic data to cooler data is available https://github.com/4dn-dcic/hic2cool tool. The cooltools used for the computation of insulation score is available at https://github.com/open2c/cooltools. The optuna tool used for the hyperparameter optimization is available at https://github.com/optuna/optuna.
